# Packaging Solutions to Extend the Shelf Life of Green Asparagus (*Asparagus officinalis* L.) ‘Vegalim’

**DOI:** 10.3390/foods10020478

**Published:** 2021-02-22

**Authors:** Stefania Toscano, Valeria Rizzo, Fabio Licciardello, Daniela Romano, Giuseppe Muratore

**Affiliations:** 1 Department of Agriculture, Food and Environment (Di3A), University of Catania, Via Santa Sofia, 100, 95123 Catania, Italy; stefania.toscano@unict.it (S.T.); dromano@unict.it (D.R.); giuseppe.muratore@unict.it (G.M.); 2 Department of Life Sciences, University of Modena and Reggio Emilia, Via Amendola 2, 42122 Reggio Emilia, Italy; fabio.licciardello@unimore.it

**Keywords:** asparagus, enzyme activity, lignin, fiber, weight loss, color, polypropylene film

## Abstract

The aim of the study was to assess, through a comparative shelf-life test, the suitability of two packaging materials, namely macro-perforated polypropylene (PP MA) and micro-perforated coextruded polypropylene (PP C), for the quality preservation of green asparagus (*Asparagus officinalis* L. ‘Vegalim’). Quality of spears was evaluated during 30 days at refrigerated storage by monitoring chemical, physical, and enzymatic parameters as well as sensory descriptors. PP C kept headspace composition close to suggested values for fresh green asparagus. Total color difference increased during the storage and it was highly correlated with chlorophyll-*a* and carotenoids, however, sensory color perception did not change significantly until 22 days of storage. PP C maintained ascorbic acid concentrations close to the initial levels, limited total phenolic compound loss to 24% (45% in PP MA), determined an increase of 72% in fiber content and small changes in lignin value; enzymatic changes were significantly inhibited. Significant sensorial differences were detected after 22 days of storage, with PP C performing better than PP MA. PP C film was confirmed as the best choice, limiting weight loss and maintaining a fresh-like appearance during 30 days of storage, thus allowing an extension in postharvest life.

## 1. Introduction

Recently, thanks to its high dietary value and the presence of vitamin C, amino acids, sterols, and fiber, as well as bioactive compounds, such as flavonoids and other polyphenolic compounds, including quercetin and cinnamic acid [1,2], asparagus (*Asparagus officinalis* L.) has been increasingly consumed [3]. Therefore, the need has arisen for effective preservation strategies that might retard quality loss, thus allowing long-distance distribution and extended shelf life. Asparagus is a highly perishable vegetable, both for its delicate structure and for its proneness to physiological alterations [4]. During storage, asparagus spear quality is reduced due to toughening, loss of water, and changes the levels of in ascorbic acid, carbohydrates, protein, and amino acids [5].

The short shelf life of asparagus is mainly due to its high respiration rate of about 60 mg CO_2_/kg × h at 5 °C [6], which continues after harvesting.

‘Vegalim’ is a male hybrid that is ideal for green asparagus production in regions with a warm or Mediterranean climate. The main characteristics are a medium-early production with high yields and excellent close tips, even in hot or warm climates. Fertile and well drained soils are preferred. This variety is bred in the Netherlands and it is suggested for green asparagus production under warm conditions, so the spear opening is delayed as temperature rises [7]. Nevertheless, the cultivar shows good adaptability to winter Mediterranean climate, so the production normally starts in January. It can also be adapted to the production of white asparagus, and spears are characterized by high weight and uniform size.

Fresh vegetables should ideally retain freshness during the distribution life, and be reasonably defect-free—thus, suitable packaging solutions should be designed to maintain the highest quality possible for the longest time. After harvest, asparagus spears are subjected to different changes, which result in the loss of quality, including the withering of spears, changes in sugar and organic acid content, chlorophyll degradation, and lignification of pericyclic fibers that results in toughening of tissue [8,9]. The above undesirable effects can be reduced by quick cooling upon harvesting, refrigeration (below 5 °C for long term storage), and storage in a modified atmosphere [10]. Sensory characteristics are used by customers as quality standards in vegetable foods; however, the food industry needs simpler methods to visualize the sensory quality achieved by producers rather than the intensities of the descriptors [11].

The degree of lignification is one of the principal factors in determining the quality of green asparagus. Lignin biosynthesis is associated with phenylalanine ammonia-lyase (PAL) and peroxidase (POD) activity in plant tissues. Being able to inhibit or regulate the activity of POD and PAL enzymes could actually improve the quality of green asparagus [12].

Senescence is also associated with the defense system that triggers the response of antioxidant enzymes and antioxidant substances naturally present in the vegetables. Antioxidant enzymes (superoxide dismutase (SOD), catalase (CAT) and peroxidase (POD), for example) are of fundamental importance in the radical detoxification process in plant tissues [13,14].

Packaging plays a key role in quality preservation of foods. With regards to fresh horticultural products, packaging may limit water vapor transmission and regulate gas exchange. In turn, these events result in the reduction of weight loss and withering degree and in the slow-down of respiration. Since vegetable senescence is associated with the extent of metabolic activities, the slow-down of respiration will result in prolonged quality maintenance and retention of bioactive components [15]. The selection of suitable packaging materials is crucial, since inappropriate packaging properties may reduce, rather than extend, the product shelf life, by determining unsuitable headspace conditions resulting in a metabolic imbalance [16].

Overall, the high economic value of this vegetable and its high perishability have raised the need to look for possible strategies to increase its shelf life, since nowadays asparagus spears are often distributed unpacked, in bunches surrounded by paperboard or plastic bands. Therefore, the aim of the current study was to assess the performance of two packaging solutions that could be applied by producers, in terms of potential for shelf-life extension of green asparagus. The study was carried out by monitoring changes in color, nutritional parameters, and sensory properties related to physiological changes, with a specific focus on the enzymatic changes occurring during refrigerated storage.

## 2. Materials and Methods

### 2.1. Biological Material

Fresh green asparagus spears (*Asparagus officinalis* L. ‘Vegalim’) were kindly provided by Asparago Sovrano Consortium (Enna, Italy).

Commercially mature spears of 28.5 ± 1.5 cm were harvested and transported to the Di3A laboratories, then undamaged and straight spears, of about 16–20 mm in diameter and ~22 cm in length with closed bracts and no visible signs of injury were selected for the study.

### 2.2. Sample Preparation and Packaging Material

Green asparagus (‘Vegalim’) were divided into 2 homogeneous batches and packaged into polypropylene macro-perforated bags (PP MA) with a piercing density of 7 holes cm^−2^ (Bemis Le Trait sas, Bourg Achard, France) and in micro-perforated coextruded PP bags (CoralifeSwaf C, Corapack s.r.l., Brenna (CO), Italy), with a row of central holes spaced 3 cm apart and having an oxygen transmission rate of 38,800 cc m^−2^ (PP C). In total, 60 bags (20 × 35 cm) were prepared using a manual sealing bar and filled with 65 ± 10 g of fresh asparagus spears. All tasters were kept under refrigerated conditions at 4 ± 1 °C and 90–95% Relative Humidity (RH) until analyses. Considering the natural decay of the product, quality parameters were monitored every 7 days and performed on three replicates for each batch; reported results were after 0 (packaging day), 8, 15, 22 and 30 days of storage.

### 2.3. Headspace Gas Composition and Color Determination

The headspace gas composition was measured as reported by Villanueva et al. [3] using a Dansensor Checkpoint (Dansensor, Ringsted, Denmark) and analyzing 10 mL of the package headspace on three replicates.

Color was measured on three spears randomly chosen from two different bags, with a Minolta portable colorimeter (CR-400 Konica Minolta, Inc., Osaka, Japan) using the CIEL*a*b* color parameters, L*, a*, and b*. The equipment was calibrated by a standard white calibration plate and using C as illumination source. Total color difference ΔE* was calculated as reported by Licciardello et al. [17].

### 2.4. Chemical Analyses

Ascorbic acid was determined according to Association of Official Agricultural Chemists (AOAC) [18]; briefly 10 g of samples were homogenized by Ultraturrax for 2 min in 40 mL extraction solution prepared by dissolving 15 g metaphosphoric acid in 40 mL acetic acid and bringing to a final volume of 500 mL with distilled water; 2 mL 2,6 dichloroindophenol diluted 1:5 was mixed with 0.5 mL extract and the absorbance was monitored at 518 nm by a Shimadzu 1601 UV-Visible spectrophotometer (Shimadzu Corp. Tokyo, Japan). The quantification was obtained by a calibration curve of suitable dilutions of standard ascorbic acid.

Total polyphenols were measured and quantified using the Folin–Ciocalteu spectrophotometric method [19] The absorbance was measured at 740 nm using a Shimadzu 1601 UV-Visible spectrophotometer (Shimadzu Corp. Tokyo, Japan), and the total polyphenol content (TPC) was then determined on the basis of a standard calibration curve generated with known amounts of gallic acid. Data were expressed as g of gallic acid equivalent (GAE) kg^−1^ of fresh weight.

Chlorophylls and carotenoids were determined according to Li and Zhang [20] and Lichtenthaler [21]. Briefly, 2 g of asparagus were homogenized with acetone and analyzed, after centrifuging, at 645 and 662 nm for chlorophyll *a* (Chl-a) and *b* (Chl-b), respectively, and at 470 nm for carotenoids.

Fiber content was determined as reported by Sothornvit and Kiatchanapaibul [22]. Samples were heated in hot water and 50% (*w*/*v*) NaOH; then, fiber was dried and weighed to calculate the percentage of fiber content, while lignin content was determined using the thioacidoglycolysis method, as proposed by Techavuthiporn and Boonyaritthongchai [23]. The absorbance was read at 280 nm using a UV-2401 spectrophotometer (Shimadzu Co., Kyoto, Japan) and the quantification was obtained using standard curves of p-coumaric acid; lignin content was expressed as g kg^−1^ of fresh weight.

All chemical analyses were performed in triplicate. Reagents and solvents were purchased from Sigma-Aldrich (Milan, Italy) and were of analytical or HPLC grade. Bi-distilled water was used throughout these tests.

### 2.5. Enzyme Analysis and MDA Content

For antioxidant enzyme assays, 0.4 g of asparagus were ground to a fine powder with liquid nitrogen and was extracted with 6 mL of extraction buffer (0.1 mol L^−1^ borate, 0.1% mercaptoethanol; pH 8.8). The homogenate was centrifuged at 10,600× *g* for 15 min at 4 °C, and the supernatant was used for enzyme activity and protein determinations.

The superoxide dismutase (SOD; EC 1.15.1.1) activity based on the measurement of inhibition in the photochemical reduction of nitroblue tetrazolium (NBT) was determined by method of Giannopolitis and Ries [24]. The catalase activity (CAT; EC 1.11.1.6) was analyzed according to Aebi [25]. The glutathione peroxidase (GPX; EC 1.11.1.7) activity was determined as described by Ruley et al. [26].

The concentration of protein was assayed using Bovine serum albumin (BSA) as a standard [27].

Phenylalanine ammonia-lyase activity (PAL; EC 4.3.1.5) was measured according to Mozzetti et al. [28]; 0.2 mL of the extract was added to 2.3 mL of reaction buffer (borate 0.1 M, 10 mM phenylalanine; pH 8.8). The mix was incubated at 40 °C and the reaction stopped, after 60 min, by addition of 0.5 mL of 5 N HCl. The absorbance was read at 290 nm. PAL activity was expressed as the mol of cinnamic acid per kg of tissue produced under the specific conditions.

Malondialdehyde content was determined based on the method used by Heath and Packer [29] and Li, et al. [30]. First, 0.5 g of asparagus was ground to a fine powder with liquid nitrogen and extracted with 1.5 mL of 5% trichloroacetic acid solution. Then, the homogenate was centrifuged at 5000× *g* for 10 min, and the supernatant was diluted to 10 mL. The same volume of 0.67% TBA was mixed with the diluted extract (2 mL). The mixture was incubated for 30 min at 100 °C in water bath and then centrifuged at 5000× *g* for 10 min. The MDA content was expressed as nmol kg^−1^ of FW.

### 2.6. Sensory Test

A trained panel consisting of five department faculty members conducted the sensory evaluations on the raw packaged spears, after refrigerated storage at 4 ± 1 °C and 90–95% relative humidity. All evaluations took place at the quality and packaging laboratory at 20 °C.

To reduce first-sample and carry-over effects, a randomized distribution was used, and different spears were randomly chosen from each packaging solution, for each assessor.

The sensory analysis test was carried out as reported by Arana et al. [11] and the procedure followed the standard ISO/IEC 17,025 [31].

Firstly, visual references were registered, and spears were evaluated for the following descriptors: ‘artichoke-shaped tip’, ‘firmness’, ‘color’, ‘fibrousness’, ‘growth of mold’, ‘odors’, ‘off-odors’, and ‘overall’. The scale intensity scores were from 0 to 5. The ‘artichoke-shaped tip’ descriptor was defined as the apical bud with a shape resembling an artichoke, and the evaluation technique consisted of the observation of the tip or bud, noticing the presence of scaly sprouts, and giving 0 to a “close tip”, meaning the highest quality; the definition for ‘firmness’ was the asparagus’ resistance to deformation by gravity, and giving 0 was the worst quality. The other descriptors were evaluated using a discontinuous scale from 0 (absence of sensation) to 5 (extremely intense) Supplementary Materials.

In order to award the final quality score of each descriptor considering its relative importance, the quality scores were multiplied by the weighting coefficients, and the ‘maximum score’ of each descriptor was then the percentage of importance assigned to it with respect to the global quality of the sample [11].

### 2.7. Statistical Analysis

The data were subjected to a two-way analysis of variance (ANOVA) as a factorial combination of packaging film × storage time. The means were compared with Tukey-test (*p* < 0.05) using CoStat release 6.311 (CoHort Software, Monterey, CA, USA). The sensory data for each attribute were submitted to one-way ANOVA using samples as factors.

## 3. Results and Discussion

### 3.1. Effect of Different Packaging and Storage Time on Respiration Rate and Nutritional Quality Parameters

As known, weight loss was significantly higher in the macro-perforated packaging than in the PP C samples: the latter material was able to limit weight loss to 0.67%, compared to 15.8% for PP MA [32]. Statistics underlined the significance of weight loss for packaging and storage as well as their interaction (Table 1) and as confirmed by data, the PP C film is suitable to slow down transpiration, thus limiting the weight loss of the samples. A previous study suggested an oxygen concentration similar to air composition (21%) and a carbon dioxide concentration in the range of 5 to 10% at 5 °C as an adequate atmosphere of storage for asparagus sprouts [33].

Of course, the only modification in headspace composition in our study took place in PP C bags, because PP C had a controlled permeability to gas, while PP MA bags were used to guarantee hygienic condition as well as product containment but allowed non- controlled gas transfer due to macro-perforation. The observed changes in headspace gas composition (Table 1) were determined both by O_2_ consumption and by CO_2_ production by green asparagus and by the gas permeability through the film.

O_2_ values were slightly below the recommended value, ranging around 18% from the 15 days of storage, followed by steady levels; however, CO_2_ stabilized around to 3.4% after 15 days. During studies of prolonged shelf life, PP C demonstrated the ability to keep the internal atmosphere close to the suggested value maintaining the good quality of fresh green asparagus.

The performance of chromatic indexes (L*, a*, b*, and ∆E) according to the analysis of variance is reported in Table 1. Packaging influenced only a^*^ (*p* ≤ 0.01), b* was influenced by storage time at *p* ≤ 0.05, while all other interactions were not statistically significant.

Considering the intricacy of color representation, it is important to remember that the model CIEL*a*b* has a wider range of color, in order to better represent the difference as well as the variation perceived by the human eyes. For this reason, a decrease in one component often corresponds to an increase in another, maintaining stable visual color [34]. During the storage time, from the start of the trial to the end, a small increase in L* values as well as in total color difference was reported. The total color difference (∆E), as expected, increased during storage despite values in the range 3 to 6 being considered quite distinguishable [34] (Table 1). The negative value of a* indicates a greater tendency towards white color [35].

Ascorbic acid content was highly influenced by storage and packaging conditions (Table 1). It is known that ascorbic acid is one of the most sensitive components and is susceptible to degradation when fresh produce is subject to unsuitable handling and storage conditions [20]. Values found on our samples, and reported in Table 2, at the beginning of the study (0.295 g kg^−1^) were in line with those reported by other authors, i.e., around 0.190–0.350 g kg^−1^ [19,20].

Samples in PP MA packaging had a quicker degradation of ascorbic acid, with a final content of 0.219 g kg^−1^, compared to samples packed in PP C which almost maintained the initial concentration, with a loss of 8% after 30 days of storage. Asparagus spears packed into PP C also showed an increase of AsAC during storage (0.349 g kg^−1^ after 22 d) according to a previous study [36]; this path can be explained as a dynamic equilibrium between the starting ascorbic acid value and the production coming from oxidative and senescence processes.

**Figure 1:**
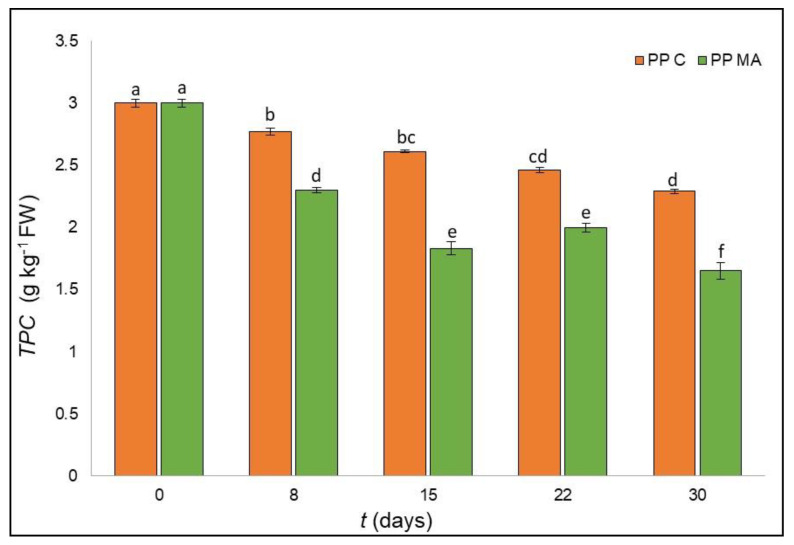
Total phenolic content of green asparagus in PP C and PP MA during 30 days of storage. Data represent the mean of three replicates ± standard deviation. Means with different letters are significantly different (*p* < 0.05) according to Tukey’s test.

The starting concentration of total polyphenols was 3.0 g kg^−1^ of gallic acid equivalent (Figure 1), higher than values found in other studies [19], reporting values in the range 0.810 to 1.74 g kg^−1^ as a function of ripening, seasonal, and environmental factors and their interactions with agronomic practices as well as the different asparagus cultivars.

Total polyphenol content (TPC) was influenced by both storage and packaging conditions tested and by their interaction (*p* < 0.001) (Table 1). TPC decreased for both packaging materials tested: samples packed in PP MA lost 45% of the initial value (1.65 g kg^−1^ after 30 d), while spears packed in PP C limited the decrease to a final value of 2.3 g kg^−1^, which corresponds to a 24% loss compared to the initial value. It is evident that PP C was able to preserve TPC content during shelf life, considering that the lowest concentration found after 30 days of storage corresponded to the amount found in PP MA after 8 days of storage (Figure 1).

The quality of asparagus spears is differently influenced by the amount of chlorophyll *a* and *b* considering that their ratio inside the chloroplast is 3:1 [37]. The values determined in the fresh product, corresponded exactly to the previous cited ratio, with a starting concentration of 667 mg kg^−1^ in Chl-*a* and 205 mg kg^−1^ for Chl-*b* (Table 2), and they were both influenced by packaging and storage as well as their interaction (Table 1). Moreover, the total chlorophyll concentration also decreased during storage, in both films tested, according to previous studies [20,38].

The Chl-*a* content decreased quickly in the first 8 days for both packaging materials, even reaching a drop of the 90% in samples packed in PP MA (Table 1), probably due the higher gas exchange and faster metabolism, followed by an increase after 15 and 22 days (Table 2), probably due to a decrease in fresh weight caused by a greater water loss of PP MA, which reached undetectable levels after 30 days of storage. In contrast, the Chl-*b* content was slightly more stable during storage, with a more evident drop-off after 22 days in PP MA then in PP C, saving an amount of Chl*-b* in the range of 45 to 80% which was related to water loss (Table 2).

Carotenoids in asparagus spears are not visible, because their color is masked by the green of chlorophylls, but they have a nutritional value if present in low concentrations [38]; their level was influenced by both storage and packaging condition as reported in Table 1. In fresh asparagus spears, the total carotenoid content was 20 mg kg^−1^ (Table 2); after 8 days of storage there was an almost complete (97%) loss in samples packed in PP MA, while samples stored in PP C limited loss to about 58.9% in the same period. Afterwards, at the end of the shelf-life study, the total carotenoid content was not detected.

Considering the possible correlations among color coordinates and the concentration of the pigments analyzed, during the period of storage, they showed a second degree polynomic model. In particular, many of them had the weakest correlation with the color parameters, with an r^2^ that did not exceed 0.65. The correlations were slightly higher for the concentrations of Chl-*b* with L* coordinate and of Chl-*a* with a* and ∆E with coefficient of determination of 0.798, 0.828, and 0.983, respectively. Interestingly, the correlation of carotenoid with ∆E had a coefficient of determination equal to 1. However, from all the parameters, ∆E could best be used to estimate the concentration of these pigments. This seems logical considering that this parameter was calculated as it simultaneously utilized the L*, a*, and b* color coordinates, as indicated by its high r^2^ values with Chl-*a* and carotenoid.

Texture is one of the main factors in determining eating and cooking quality of fresh asparagus and excessive fiber is a disagreeable quality characteristic. Spear lignification, of the fiber ring and the vascular bundles, in asparagus causes toughening [39]. As reported in Table 3, the analysis of variance showed that packaging and the storage time had a significant (*p* < 0.001) effect for both fiber and lignin parameters.

**Figure 2:**
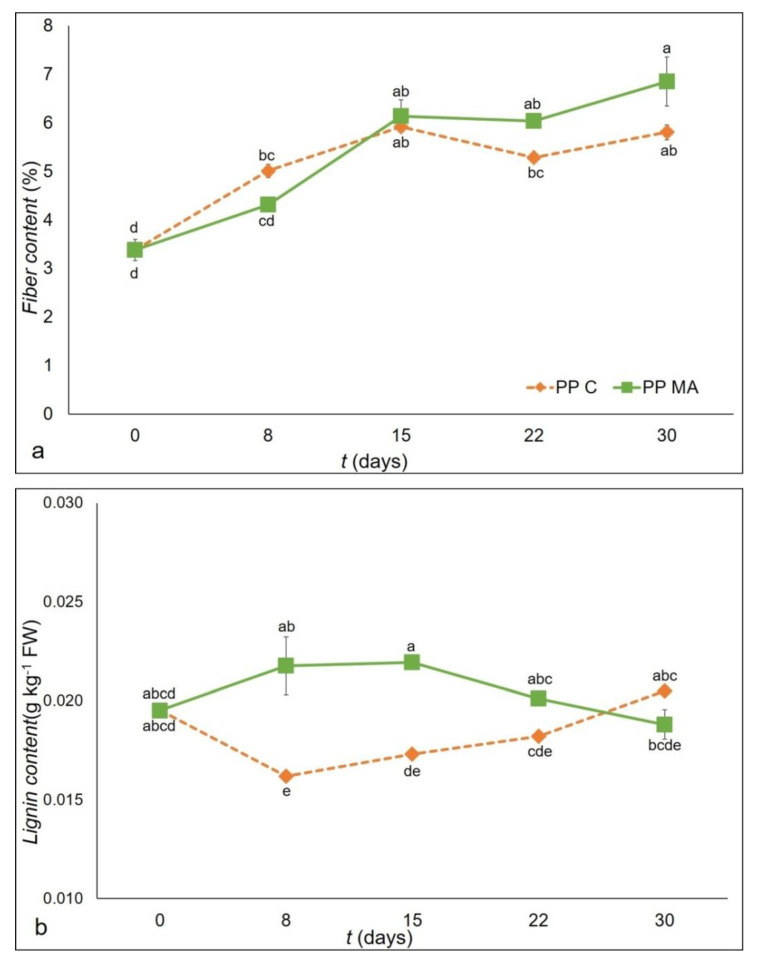
Fiber content (**a**) and lignin content (**b**) as affected by the ‘packaging material × storage time’ interaction of green asparagus during 30 days of storage in PP C and PP MA. Data are the average of three replicates ± standard deviation. Means with different letters are significantly different (*p* < 0.05) according to Tukey’s test.

As expected, an increase in fiber content was observed for samples in both plastic films tested (Figure 2a). The initial fiber content was 3.38%, a percentage slightly higher compared to values reported in literature, but this is probably a specific characteristic of the ‘Vegalim’ genotype; among packaging, samples packed in PP C showed an increase of 72% after 30 days of storage (Figure 2a), reaching a fiber content of 5.81% against the final value of 6.85% recorded by PP MA that increased the starting value by around 102%.

Lignin content is one of the components of the sclerenchyma cell of asparagus fiber and it is considered as the main freshness attribute of asparagus. Increase in lignin levels during storage results in the toughening which occurs in the asparagus a few days after harvest [4]. The initial lignin value was 0.019 g kg^−1^ in line with data reported by An et al. [4]; changes in lignin content were evaluated during storage and reported in Figure 2b. Changes of lignin content of packaged asparagus were symmetrical; in PP MA there was an increase that reached a value of 0.023 g kg^−1^ after 8 days of storage, with a new decrease at the end of the study of 0.018 g kg^−1^; while the performance of PP C showed a decrease after 8 days of storage to 0.016 g kg^−1^ and then an increase to values similar to those recorded at the start of the experiment, despite indicating small changes during the 30 days of the study, with a final value of 0.02 g kg^−1^.

### 3.2. Effect of Different Packaging and Storage Timse on Enzymes and MDA Content

**Figure 3:**
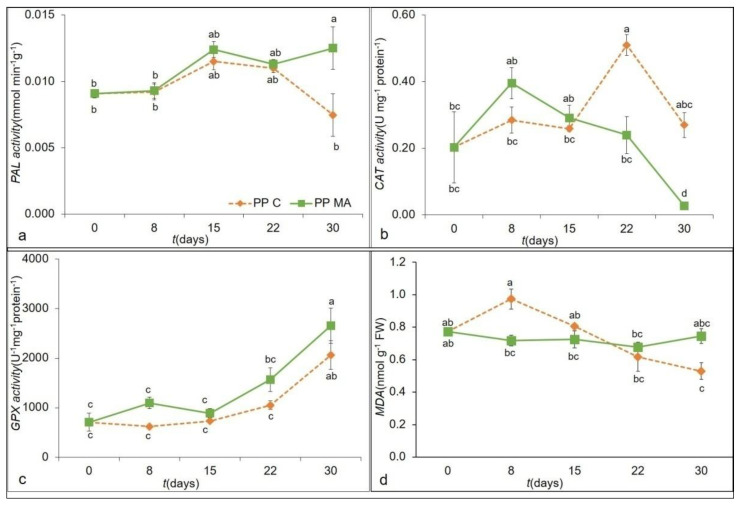
PAL activity (PAL) (**a**), catalase (CAT) (**b**), glutathione peroxidase (GPX) (**c**), and malondialdehyde (MDA) (**d**). as affected by the ‘packaging material × storage time’ interaction of green asparagus during 30 days of storage in PP C and PP MA. Data are the average of three replicates ± standard deviation. Means with different letters are significantly different (*p* < 0.05) according to Tukey’s test.

The phenylalanine ammonia-lyase (PAL), is a primary enzyme of the phenylpropanoid pathway that produces anthocyanins, flavonoids, and lignin, and it is the key enzyme responsible for lignification of asparagus spears. It is also the key enzyme in the synthesis of phenolic compounds, which can decompose phenylalanine into phenols. As the initial step of the phenylpropanoid pathway, PAL mainly breaks down phenylalanine into cinnamic acid, a key intermediate in the pathway of lignin production. Controlling its activity can be one of the effective ways to delay hardening, as reported by Toscano et al., [40], who reduced the PAL activity by treatment with ammonium sulfate (2 mmol L^−1^). The analysis of variance (Table 3) showed that packaging and the storage time had a significant (*p* < 0.001) effect on the PAL activity. In PP MA, PAL activity showed a significant increase compared to PP C at the end of the trial (30 days), with an increase of 40% (Figure 3a).

Thus, according to the data obtained, the activity of PAL enzyme could be significantly inhibited by packaging and storage into PP C. Indeed, to maintain some qualitative characteristics of green asparagus, it could help the inhibition or regulation of this enzymatic activity [41].

In addition to PAL, other enzymes regulate the process of lignification of spears. Apel and Hirt, have shown that to reduce the effects of oxidative stress, plants have evolved efficient complex enzymatic and non-enzymatic systems, such as glutathione, ascorbic acid, carotenoids, and reactive oxygen species (ROS) scavenging enzymes (SOD, GPX, CAT, and APX) [42]. The effect of packaging and storage time and their interactions on the CAT, SOD, and GPX in the samples are showed in Table 3. The analysis of variance (Table 3) showed that the packaging and the storage time also had a significant (*p* < 0.01) effect on the CAT activity. As shown in Figure 3b, during the storage time, significant differences in CAT activity between the packaging materials emerged after 22 days of storage, with an increase of 53% in PP C compared to PP MA. This parameter declined throughout time, reaching values close to 0 in the PP MA treatment at the end of the trial.

No significant differences in the SOD activity among the packaging and the storage were detected (Table 3). The SOD activity was significantly affected only by packaging. Toivonen and Sweeney, in a study on two genotypes of broccoli, showed that a delay of senescence was correlated to increase of SOD activities [43]. From the results of our study, it can be hypothesized that PP C might delay the senescence of asparagus spears by means of regulating the antioxidant enzyme systems.

Storage time and its interaction with packaging significantly (*p* < 0.01 and *p* < 0.001, respectively) influenced the GPX activity, as evidenced by the ANOVA (Table 3). Our results showed that the GPX activity increased at the end of the trial in both films with an increase of 65% in PP MA and 73% in PP C compared to the initial value (Figure 3c). Asparagus spears treated with PP MA showed, in general, a higher GPX activity than PP C, which might have resulted in efficient conversion of the superoxide anion radical to H_2_O_2_ then to H_2_O [44].

However, SOD and GPX enzyme activities in asparagus are diminished with the lengthening of postharvest time [4], which results in the accumulation of hydrogen peroxide (H_2_O_2_) in organism [45,46].

The thiobarbituric acid-reactive substance (MDA) is one of final products of polyunsaturated fatty acid oxidation of the cell membrane, resulting as product of membrane lipid peroxidation, which is caused by accumulation of reactive oxygen species and it has been used as a direct indicator of membrane injury. In our study the MDA content remained constant during the storage time in both treatments (Figure 3d). After only 8 days the MDA content of asparagus in PP C was significantly higher (26%) than that stored in PP MA. In several studies of fruit and vegetable shelf life, a continuous increase in MDA content was also observed; for post-harvest broccoli it was explained [47,48] that senescence is related to lipid peroxidation (MDA content) resulting to disintegration of the cell membrane.

### 3.3. Sensorial Test

Table 4 shows the mean scores of the significant descriptive sensory analysis attributes of ‘Vegalim’ green asparagus in the two different packaging materials. As can be observed, no significant difference between treatments was detected in the first 15 days of storage for any of the eight quality attributes considered. After 22 days of storage the descriptors which recorded the most significant differences were turgidity, firmness, color, and total acceptability. These scored better values for PP C than for PP MA, with artichoke-shaped tip and odors being identified as different. It is noteworthy that the statistically significant changes highlighted by instrumental color parameters did not correspond to perceived color differences until 22 days of storage. This could be due to samples’ variability and to the small, though significant, differences recorded by the colorimeter. At the end of the shelf-life study, the main differences were, again, in firmness, color, turgidity, and total acceptability, the same four parameters which showed significant differences in the previous sampling.

In this last sampling (30 days) the judges’ assessments were similar to the previous ones for PP MA, while the values of samples in PP C were noticeably worse. The worst attributes, mold growth and off-odors, were not detected by judges, proving the genotype ‘Vegalim’ suitable for prolonged refrigerated storage in both packaging materials tested.

Overall, the comparison between the two packaging materials showed that a film with controlled gas permeation rather than a macro-perforated film is more suitable for quality maintenance of fresh asparagus spears by reducing water vapor transmission, thus creating a saturated moisture atmosphere which minimizes transpiration and consequent withering. Similarly, the PP C film was able to generate a suitable headspace, close to values suggested in literature, which resulted from the combination of the vegetable metabolism (O_2_ uptake and CO_2_ emission) and film permeability (CO_2_ flowing outwards and O_2_ inwards), following partial pressure gradients. The resulting passive atmosphere has positive effects on the metabolism of the vegetable, as was confirmed by the better retention of bioactive components and highest sensory scores.

## 4. Conclusions

Despite many previous papers studying different postharvest treatments for maintaining the quality of asparagus spears, and focusing on changes in packaging materials and storage conditions such as temperature or relative humidity, or modifying the atmosphere, changing oxygen, carbon dioxide, and nitrogen percentage, few of them have considered a prolonged shelf life as we did in our study. A longer shelf life has a double-positive effect both for limiting food waste and extending the economic value for the presence on the market. Since, nowadays, asparagus spears are often distributed unpacked, in bunches surrounded by paperboard or plastic bands, it is relevant to assess the potential of packaging materials commonly used for fruits and vegetables (polyethylene, low density polyethylene, and polypropylene) for shelf-life extension. PP C film was confirmed as the best choice between our tested plastic films. PP C package could effectively limit water loss and maintain the fresh appearance of fresh asparagus spears during storage, thus allowing an extension in postharvest life. It controlled the weight loss and kept, naturally, the internal atmosphere close to suggested values for fresh green asparagus according to literature data. Also, from a chemical point of view, PP C had the best performance. Asparagus spears packed in PP C could slow down carotenoid loss, reduce PAL, GPX and MDA activity after 30 days of refrigerated storage and improve sensorial qualities compared with PP MA. In PP MA, the observed AsAC, TPC, fiber, and lignin concentrations are partially determined by a significant level of dehydration, as proved by weight loss, which was almost negligible in PP C. In other words, the observed difference would be even greater if we considered those parameters on dry weight basis. Mold growth and off-odors were not detected, proving ‘Vegalim’ asparagus as a suitable variety for prolonged refrigerated storage. Providing this genotype with a suitable packaging such as PP C would greatly improve its potential for distribution by retarding senescence and maintaining higher quality parameters.

Research on packaging materials is nowadays highly focused on biodegradable materials, especially for fresh products packaging. However, the use of conventional plastic materials still offers higher convenience, especially when mono-material films are concerned. Indeed, PP films can be effectively recovered and recycled in the current waste management systems, while biodegradable films, to date, represent an issue for the current composting facilities. PP C film represents a viable strategy for shelf life extension of green asparagus and it can thus represent a reference material for future evaluation of biodegradable/compostable materials once cost, performance, and waste management aspects are improved.

## Figures and Tables

**Table 1:** Effects of “packaging (P)” and “storage time (S)” on qualitative traits, weight loss (%); carbon dioxide (CO_2_) and oxygen (O_2_) headspace concentrations (%); color parameters (L*, a*, b*, and ∆E); ascorbic acid content (AsAC) (g kg^−1^ FW); total polyphenol content (TPC) (g kg^−1^ FW); chlorophyll-*a* (Chl-*a*); chlorophyll-*b* (Chl-*b*); and total carotenoids (Car) (mg kg^−1^ FW) on green asparagus spears (*Asparagus officinalis* L. ‘Vegalim’). Two packaging materials were considered—PP C (polypropylene micro-perforated bags) and PP MA (polypropylene macro-perforated bags).

		Qualitative Trait											
			PP C†										
		WL (%)	CO_2_ (%)	O_2_ (%)	L*	a*	b*	∆E	AsAC (g kg^−1^ FW)	TPC (g kg^−1^ FW)	Chl-a (mg kg^−1^ FW)	Chl-b (mg kg^−1^ FW)	Car (mg kg^−1^ FW)
Packaging (P)													
	PP C	0.25 ± 0.06 b	3.62 ± 1.07	17.86 ± 1.61	49.41 ± 3.59	−10.57 ± 0.85 a	26.00 ± 1.55	3.31 ± 1.54	0.29 ± 0.01 a	2.63 ± 0.07 a	200.21 ± 63.6 a	207.76 ± 15.14 a	6.19 ± 0.78 a
	PP MA	5.78 ± 1.14 a	-	-	48.28 ± 3.00	−11.78 ± 0.81 b	26.50 ± 1.39	2.00 ± 0.96	0.26 ± 0.01 b	2.16 ± 0.13 b	183.36 ± 65.34 b	153.45 ± 29.17 b	5.03 ± 1.02 b
Storage time (S)													
	0	0.00 ± 0.00 d	1.00 ± 0.02	21.00 ± 0.10	48.31 ± 1.65	−11.61 ± 0.46	26.95 ± 0.20 a	0.00 ± 0.00	0.29 ± 0.01 a	3.00 ± 0.02 a	667.42 ± 0.98 a	205.38 ± 0.18 ab	20.01 ± 0.00 a
	8	1.43 ± 0.64 cd	6.50 ± 2.60	14.13 ± 4.90	46.89 ± 1.31	−10.95 ± 0.47	25.03 ± 0.53 b	2.87 ± 1.58	0.27 ± 0.00 b	2.54b ± 0.10	88.98 ± 11.15 c	239.20 ± 27.89 a	4.45 ± 1.75 b
	15	3.11 ± 1.37 bc	3.80 ± 0.81	17.91 ± 0.65	47.52 ± 1.20	−10.71 ± 0.19	25.50 ± 0.50 b	2.12 ± 1.16	0.25 ± 0.01 b	2.22 ± 0.17 c	87.28 ± 7.89 c	200.10 ± 8.28 b	1.70 ± 0.39 c
	22	4.58 ± 1.96 ab	3.40 ± 0.43	18.23 ± 0.51	49.96 ± 0.98	−11.44 ± 0.25	26.96 ± 0.17 a	1.90 ± 1.61	0.31 ± 0.02 a	2.23 ± 0.11 c	112.93 ± 9.53 b	103.54 ± 20.11 d	1.88 ± 0.67 c
	30	5.95 ± 2.46 a	3.40 ± 0.45	18.03 ± 0.30	51.56 ± 0.92	−11.19 ± 0.61	26.82 ± 0.18 a	3.71 ± 1.66	0.25 ± 0.01 b	1.98 ± 0.15 d	2.30 ± 1.57 d	154.82 ± 69.58 c	0.00 ± 0.00 d
(P)		***			NS	**	NS	NS	***	***	***	**	^***^
(S)		***			NS	NS	*	NS	***	***	***	***	^***^
(P) x (S)		***			NS	NS	NS	NS	***	***	***	***	^***^

† Headspace carbon dioxide (CO_2_) and oxygen (O_2_) concentrations were monitored only on polypropylene micro-perforated bags (PP C), because PP MA (polypropylene macro-perforated bags) allowed non-controlled gas transfer. The levels of significance were represented by *p* > 0.05. NS, not significant; * significant at *p* < 0.05; ** significant at *p* < 0.01; *** significant at *p* < 0.001 according to Tukey’s test.

**Table 2:** Effect of the “packaging (P) x storage time (S)” significant interaction on ascorbic acid content (AsAC), chlorophyll-*a* (Chl-*a*), chlorophyll-*b* (Chl-*b*), and carotenoid (Car) in PP C (micro-perforated polypropylene bags) and PP MA (polypropylene macro-perforated bags).

	PP C	PP MA	PP C	PP MA	PP C	PP MA	PP C	PP MA
Storage Time (d)	AsAC (g kg^−1^FW)		Chl-*a* (mg kg^−1^FW)		Chl-*b* (mg kg^−1^FW)		Car (mg kg^−1^FW)	
0	0.295 ± 0.28 bA		667 ± 30 aA		205 ± 15 bA		20 ± 0.03 aA	
8	0.266 ± 0.19 bA	0.269 ± 0.15 abA	111 ± 15 bcA	69 ± 18 bB	185 ± 29 bcB	298 ± 37 aA	8.3 ± 0.5 bA	0.6 ± 0.06 cB
15	0.273 ± 0.08 bA	0.231 ± 0.12 bcB	89 ± 26 cA	88 ± 16 bA	194 ± 19 bcA	210 ± 22 bA	0.6 ± 0.00 cB	1.1 ± 0.12 cA
22	0.349 ± 0.05 aA	0.270 ± 0.20 abB	133 ± 15 bA	96 ± 11 bB	150 ± 10 cA	59 ± 9 cB	0.4 ± 0.00 dB	3.4 ± 0.11 bA
30	0.276 ± 0.06 bA	0.219 ± 0.03 cB	5 ± 0.5 d	n.d.	113 ± 27 cA	189 ± 32 bA	n.d.	n.d.

Values are means ± SD of four replicates (*n* = 4). Different lower-case letters within the column indicate statistically significant differences among storage times according to Tukey’s test at the significance level (*p* < 0.05); different capital letters indicate statistically significant differences according to Tukey’s test at the significance level (*p* < 0.05). n.d.; not detectable.

**Table 3:** Effects of “packaging” and “storage time” on fiber (%), lignin content (g kg^−1^ FW), phenylalanine ammonia-lyase (PAL) activity (mmol min^−1^ g^−1^), catalase (CAT), glutathione peroxidase (GPX), and superoxidedismutase (SOD) activity (U mg^−1^ protein^−1^), and malondialdehyde content (MDA) (nmol g^−1^ FW) on green asparagus spears (*Asparagus officinalis* L. ‘Vegalim’). Two packaging materials were considered—PP C (polypropylene micro-perforated bags) and PP MA (polypropylene macro-perforated bags).

		Fiber (%)	Lignin (g kg^−1^ FW)	PAL (mmol min^−1^ g^−1^)	CAT (U mg^−1^ protein^−1^)	SOD (U mg^−1^ protein^−1^)	GPX (U mg^−1^ protein^−1^)	MDA (nmol g^−1^ FW)
Packaging (P)								
	PP C	4.98 ± 0.15 b	0.018 ± 7.9 10^−5^ b	0.0096 ± 0.00 b	0.31 ± 0.03 a	25.29 ± 7.7	1034.66 ± 154.9 b	0.74 ± 0.05
	PP MA	5.64 ± 0.36 a	0.021 ± 7.9 10^−5^a	0.0109 ± 0.00 a	0.24 ± 0.04 b	27.20 ± 7.4	1383.82 ± 203.8 a	0.73 ± 0.02
Storage time (S)								
	0	3.38 ± 0.14 c	0.014 ± 0.02	0.009 ± 0.00 b	0.23 ± 0.05 ab	81.22 ± 5.4 a	708.83 ± 115.6 c	0.77 ± 0.02 ab
	8	4.66 ± 0.18 b	0.019 ± 0.00	0.009 ± 0.00 b	0.34 ± 0.04 a	12.39 ± 1.7 b	859.66 ± 119.0 bc	0.85 ± 0.07 a
	15	6.03 ± 0.16 a	0.020 ± 0.00	0.012 ± 0.00 a	0.028 ± 0.02 ab	10.54 ± 2.0 b	810.98 ± 57.5 bc	0.77 ± 0.03 ab
	22	5.66 ± 0.17 a	0.019 ± 0.00	0.011 ± 0.00 a	0.37 ± 0.01 a	8.16 ± 1.0 b	1308.92 ± 163.2 b	0.65 ± 0.04 b
	30	6.33 ± 0.33 a	0.020 ± 0.00	0.010 ± 0.00 ab	0.15 ± 0.06 b	18.93 ± 0.9 b	2357.81 ± 244.2 a	0.64 ± 0.06 b
(P)		*	***	**	**	NS	**	NS
(S)		***	NS	***	***	***	***	***
(P) x (S)		***	***	***	*	NS	**	**

NS, not significant; *significant at *p* < 0.05; ** significant at *p* < 0.01; *** significant at *p* < 0.001.

**Table 4:** Mean scores of the significant sensory attributes of asparagus in different packaging materials—PP C (micro-perforated coextruded PP film) and PP MA (macro-perforated PP film) during 30 days of storage.

Storage Time (d)	Attribute	Packaging	
		*PP C*	*PP MA*
0	No significant differences		
8	No significant differences		
15	No significant differences		
22	Artichoke-shaped tip	1.8 b	4.2 a
	Firmness	5.0 a	2.4 b
	Color	4.0 a	1.8 b
	Turgidity	4.0 a	2.0 b
	Odors	3.6 a	1.2 b
	Total acceptability	3.6 a	1.2 b
30	Firmness	4.2 a	2.4 b
	Color	4.0 a	2.6 b
	Turgidity	4.0 a	2.4 b
	Total acceptability	4.0 a	2.2 b

∗ Values marked with different letters in the same row are significantly different (*p* < 0.05) according to the Tukey’s test.

## Data Availability

Main data are contained within the article or supplementary material; further data presented in this study are available on request from the corresponding author.

## Supplementary Materials

The following are available online at https://www.mdpi.com/2304-8158/10/2/478/s1.
