# Supplementary material for: Packaging Solutions to Extend the Shelf Life of Green Asparagus (Asparagus officinalis L.) ‘Vegalim’
Source: Foods. 2021 Feb 22;10(2):478. doi: 10.3390/foods10020478 (PMC7926684; doi:10.3390/foods10020478)
Supplement: Supplementary file 1 [file foods-10-00478-s001.pdf]

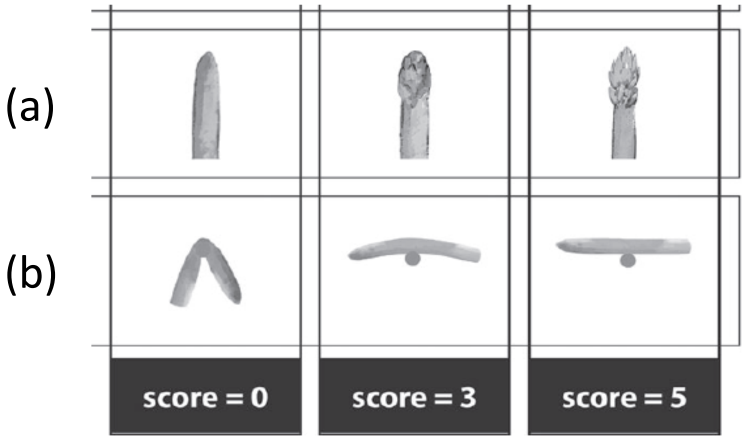

Visual references for evaluation of descriptors: (a) ‘artichoke-shaped tip’ and (b) ‘firmness’ [11]

ARTICHOKE-SHAPED TIP: 0 □ 3 □ 5 □

FIRMNESS: 0 □ 3 □ 5 □

Sign according the following scale:
‘absence’ (0), ‘very weak’ (1), ‘weak’ (2), ‘medium’ (3), ‘intense’ (4) and ‘very intense’ (5)

COLOUR 0 1 2 3 4 5

TURGIDITY 0 1 2 3 4 5

MOULDS 0 1 2 3 4 5

OFF-ODOURS 0 1 2 3 4 5

ODOURS 0 1 2 3 4 5

TOTAL ACCEPTABILITY 0 1 2 3 4 5
